# Green Synthesis of Zinc Oxide Nanoparticles Using Aqueous Extract of *Deverra tortuosa* and their Cytotoxic Activities

**DOI:** 10.1038/s41598-020-60541-1

**Published:** 2020-02-26

**Authors:** Yasser A. Selim, Maha A. Azb, Islam Ragab, Mohamed H. M. Abd El-Azim

**Affiliations:** 1 Faculty of Specific Education, Zagazig University, Zagazig, 44519 Egypt; 2 Botany Department, Faculty of Science, Zagazig University, Zagazig, 44519 Egypt; 3 Faculty of Science and Arts, Riyad Alkhabra, Qassim University, Qassim, Saudi Arabia; 4 Chemistry Department, Faculty of Science, Zagazig University, Zagazig, 44519 Egypt

**Keywords:** Drug development, Chemical safety

## Abstract

In recent years, there is a growing interest towards the green synthesis of metal nanoparticles, particularly from plants; however, yet no published study on the synthesis of ZnO.NPs using the *Deverra tortuosa* extract. Through this study, zinc oxide nanoparticles (ZnO.NPs) have been synthesized based on using the environmentally benign extract of the aerial parts of *D. tortuosa* as a reducing and capping agent. ZnO.NPs synthesis was confirmed using UV-Visible (UV-Vis) spectroscopy, Fourier Transform Infrared Spectroscopy (FTIR), X-ray Diffraction (XRD) and High Resolution-Transmission Electron Microscope (HR-TEM). The qualitative and quantitative analyses of plant extract were done. The potential anticancer activity was *in vitro* investigated against two cancer cell lines (human colon adenocarcinoma “Caco-2” and human lung adenocarcinoma “A549”) compared to their activities on the human lung fibroblast cell line (WI38) using the MTT assay. Both the aqueous extract and ZnO.NPs showed a remarkable selective cytotoxicity against the two examined cancer cell lines.

## Introduction

Nowadays, nanotechnology is expected to be the basis of many biotechnological innovations in the 21^st^ century and regarded as the upcoming industrial revolution. Nanomaterials have been called ‘a wonder of modern medicine’ and elicited much interest over the past few decades^1^. Nanomaterials are of great importance because of their superior physicochemical and biological properties over their bulk phase. The size of these nanostructured materials (1–100 nm) offers a higher surface to volume ratio which led to high surface reactivity^2^. This distinct property allowed them to be utilized in vast applications in many fields ranging from material science to biotechnology. Nanobiotechnology is the merge between biotechnology and nanotechnology for developing biosynthetic and ecofriendly technology for the synthesis of nanomaterials^3^.

Progress in utilizing inorganic nanoparticles for biomedical applications taking into the account the environmental aspect stimulated the need to synthesize them using the green chemistry strategies *via* biological systems^4,5^. Various studies suggested that plants seem to be the superior candidate and are proper for large scale biosynthesis of nanoparticles where the rate of synthesis is faster than that in the case of other organisms. In addition, the nanoparticles produced through plants are more various in shape and size in comparison with those produced by other organisms *such as bacteria, fungi and algae*^6^. Forasmuch, many bioactive constituents in plants such as alkaloids, terpenoids, flavonoids, amino acids, enzymes, vitamins, proteins, and glycosides could also be a participant in bioreduction, formation and stabilization of the metal nanoparticles^7–9^.

*Deverra tortuosa* is a wild perennial bushy plant growing naturally in sandy and stony plains^10^. This plant is widespread in deserts of the Arabian ecoregion (Saudi Arabia, Palestine, Egypt, Libya and Tunisia). This plant Synonym is *Pituranthos tortuosus* (Desf.) and known in Arabic as Shabat El-Gabal or Qozzaah. As a member of Apiaceae family, *D*. *tortuosa* possesses a characteristic pungent or aromatic scent. The presence of important bioactive compounds in different parts of the plant; flavonoids, terpenoids, glycosides, essential oil, furanocoumarins and unsaturated sterols has been previously published^11–13^. The plant is often used by local citizens in traditional medicine as analgesic, carminative, diuretic, antiasthmatic and to relieve stomach pain and against intestinal parasites^14,15^. It has been found to be pharmacologically active to treat asthma, hepatitis, fever, rheumatism, diabetes, digestive difficulties and to regulate menstruation^12,16^. Also, it was reported to be used as an edible food to treat hypertension, against constipation and in the case of bites, and been used for grazing and fuel wood^17,18^. Another possible application could be its utilization in the nanobiotechnology field.

Among the metal nanoparticles ZnO.NPs are interesting due to its impressive properties from which the wide band gap, large binding energy and high piezoelectric property^19^. ZnO.NPs which can exhibit a wide variety of nanostructures are believed to be biosafe, nontoxic and biocompatiable, been used in various technologies and industries such as optoelectronics, piezoelectric and magnetic sensors, biodiagnosis, biological labelling, ceramic and rubber processing, environmental protection, biology and medicinal industry^20–22^. Chemically, the surface of ZnO is rich in -OH groups, which permit ZnO to slowly dissolve in both acidic (e.g., the tumor cells and tumor microenvironment) and strong basic conditions. Based on this property, ZnO.NPs have gained immense interest in biomedical^23^. The extended application of ZnO.NPs in medicine as anti-angiogenesis, antiplatelet agents, anti-inflammatory, dental materials, cosmetics, drug and gene delivery, have made ZnO.NPs a promising anticancer agent^24,25^.

## Materials and Methods

### General

All chemicals of high grade of purity were obtained from Sigma-Aldrich (St. Louis, MA, USA).All solutions were prepared with double distilled water. Analyses, qualitative and quantitative measurements involved the use of the following tools and systems: WARING COMMERCIAL Lab Blender (Dynamics Corp. of America, New Hartford, CT, USA), Hei-VAP Rotary evaporator (Heidolph, Germany),1260 Infinity II LC System (Agilent, USA),Ultrasonic homogenizer model 150/vt (Biologics Inc. Cary, North Carolina), UV-Visible Spectrophotometer (JASCO, Japan), FT/IR-4100 Spectrometer (JASCO, Japan), X’Pert PRO X-ray Diffraction System (PANalytical, Netherland), JEM-2100 High Resolution Transmission Electron Microscope (JEOL Ltd, Japan), Cellstar 96-well plate (Greiner Bio One International, Austria) and MR-96A microplate reader (Shenzhen Mindray Bio-Medical Electronics Co., Ltd, China).

### Plant collection

The aerial parts of *D. tortuosa* were collected in July, 2017 from a natural ecosystem (30°20′47.8″N 31°37′51.1″E) at the Belbis City – 10^th^ of Ramadan desert road, Sharqia governorate, Egypt and were identified by Prof. Dr. Hussien Abd El-Basset, Professor of Plant Taxonomy, Faculty of Science, Zagazig University and a voucher specimen was deposited in the herbarium of Botany Department, Zagazig University, Egypt.

### Plant extraction

Plants were washed several times with double distilled water to remove any debris or particulates then shade dried at room temperature. Aerial parts were finely ground into a fine powder. The aqueous extract was prepared by the cold maceration method^26^. The plant powder (50 g) was soaked in 1 L of distilled water, kept in a shaker at 20 °C for 24 h for continuous agitation at 100 rpm for thorough mixing. Then the extract was filtered and stored at −4 °C for further investigations.

### Phytochemical screening

Preliminary phytochemical screening of the extract was carried out to identify the active constituents, using standard methods^27^.

### HPLC analysis

A high-performance liquid chromatography (HPLC) was used to detect, identify and quantify a number of phenolic compounds in the extract using an Agilent 1260 seriesfollowing a modified method by Wu *et al*.^28^. The separation was carried out using C18 column (4.6 mm ×250 mm i.d., 5 μm). The column temperature was maintained at 35 °C. The mobile phase consisted of water (A) and acetonitrile (B) at a flow rate 1 ml/min. The mobile phase was programmed consecutively in a linear gradient as follows: 0 min (80% A); 0–5 min (80% A); 5–8 min (40% A); 8–12 min (50% A); 12–14 min (80% A) and 14–16 min (80% A). The multi-wavelength detector was set at 280 nm. The injection volume was 10 μl for each of the sample solutions.

### Green synthesis of zinc oxide nanoparticles

Biogenic synthesis of ZnO.NPs was carried out according to the method of Elumalai *et al*.^29^ with modifications. The crude plant extract (about 25 mL) was heated (60–80 °C) on a magnetic stirrer. When the temperature of the extract reached 60 °C, 2.5 g of zinc nitrate hexahydrate (Zn(NO_3_)_2_.6H_2_O) was added and left for about 1 h till a white precipitate appeared. This mixture then was left overnight in a hot air oven at 60 °C or till a creamy paste formed. This paste was collected and washed several times with a solution of distilled water: Ethanol (3:1). Afterwards, the collected paste was transferred to a ceramic crucible cup and heated in furnace at 400 °C for 2 h. The resultant white powder stored in an airtight container for characterization.

### Characterization of ZnO.NPs

The ZnO.NPs initially analyzed by using the Rigol ultra-3660 UV-vis spectroscopy within the range 200–800 nm. Then FTIR was used to identify the functional groups and various phytochemical constituents involved in the reduction and stabilization of the synthesized nanoparticles. FTIR was carried out using the attenuated total reflectance (ATR) mode with a Jasco FTIR 4100 spectrophotometer (Japan). The results recorded in the range of 4000–400 cm^−1^. The powdered sample was subjected to a CuKα_1_-X Ray diffractometer radiation (λ = 1.5406 A°) operating at 40 kV and 30 mA with 2θ ranging from 30°–140° to confirm the presence of ZnO and analyze the crystallite structure and size. ZnO nanopowder was suspended in ethanol, sonicated then coated onto a copper grid and allowed to dry and examined by JEOL-2100 HR-TEM.

### Cytotoxic activity

#### Cell lines

Human colorectal epithelial adenocarcinoma “Caco-2”, human lung epithelial carcinoma “A549”) and the normal human lung fibroblast cell line (WI38) were procured from tissue culture Lab in VACSERA Institute, Agoza, Egypt (The holding company for biological products and Vaccines).

#### Cell culture and MTT assay

To evaluate the cytotoxicity of the aqueous plant extract and ZnO.NPs, the MTT (3-(4,5- dimethylthiazol-2-yl– 2,5-diphenyl tetrazolium bromide) colorimetric assay was performed in 96-well plates^30^. The whole procedure was maintained under sterile conditions *via* the use of a laminar air-flow cabinet, following culturing and sub-culturing technique adopted by Thabrew *et al*.^31^.

## Results

### Phytochemical study

All results of phytochemical analysis are showed in Table 1. In the present study, the aqueous fraction from the crude extract showed positive results for triterpenes and/or steroids as measured by the Liebermann-Burchard reaction. It was found that *D. tortuosa* aqueous extract contained polyphenols and flavonoids, which may be responsible for the biological activities found.

**Table 1:** Phytochemical screening of *D. tortuosa* extract.

Test	Result
Alkaloids	++
Anthraquinone	−
Coumarins	+++
Flavonoids	++
Glycosides	+++
Saponins	−
Steroids	++
Tannins	+++
Terpenoids	++

Highly positive ‘+++’, Moderate ‘++’, Negative ‘−’.

### HPLC analysis

**Figure 1:**
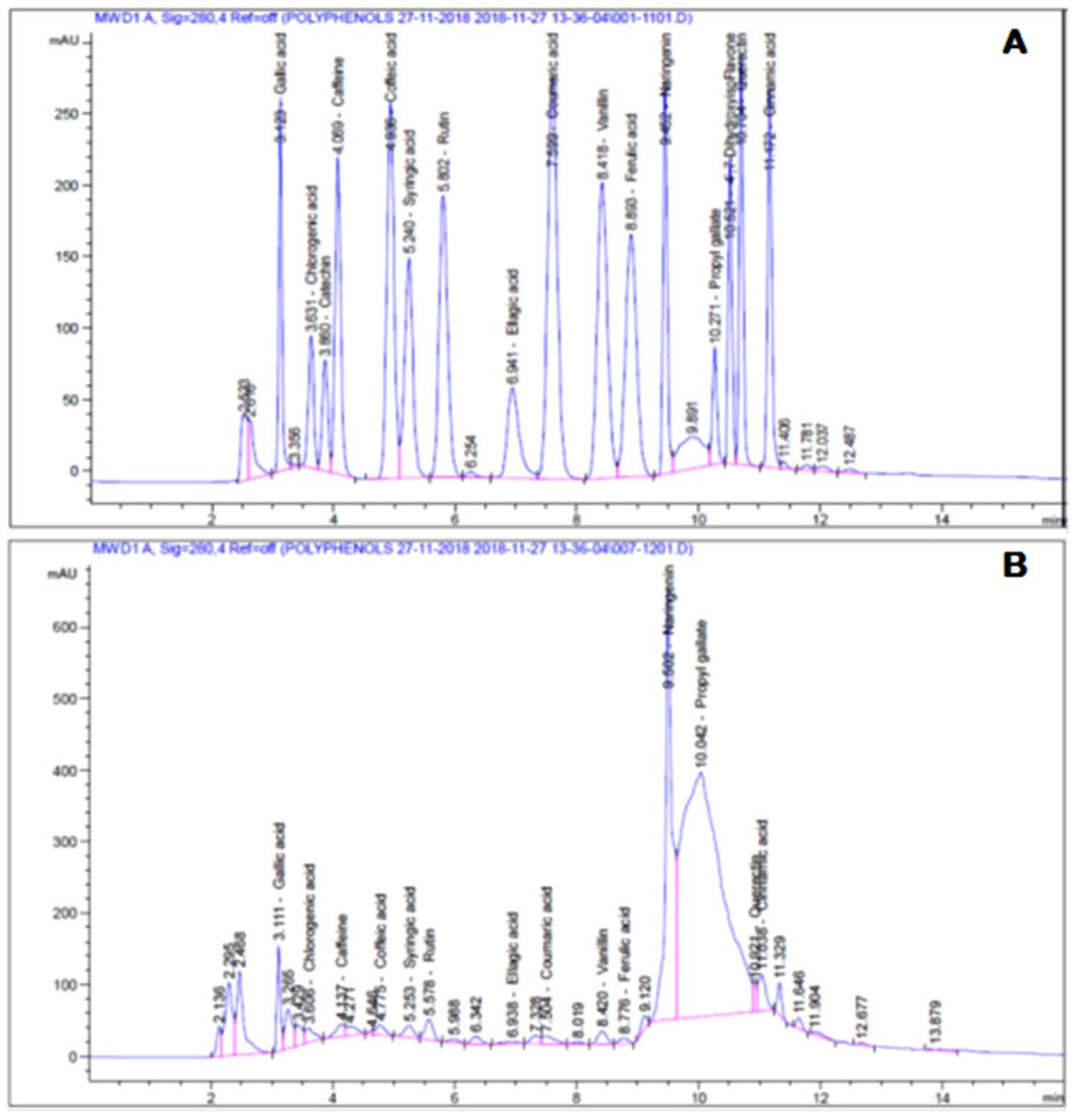
HPLC chromatogram: (**A**) Standard mixture of polyphenolic compounds; (**B**) Aqueous extract of *D. tortuosa* aerial parts.

**Figure 2:**
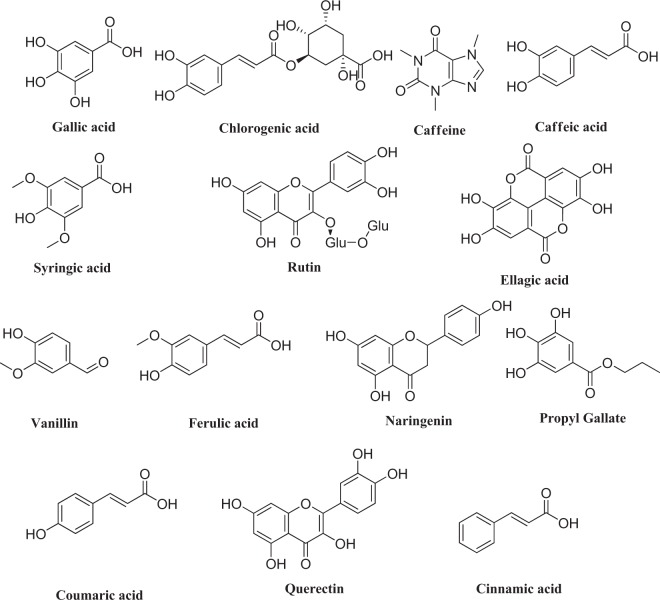
Chemical structures of polyphenolic compounds of *D. tortuosa* aerial parts.

HPLC analysis indicate the presence of Gallic acid, Chlorogenic acid, Caffeine, Coffeic acid, Syringic acid, Rutin, Ellagic acid, Coumaric acid, Vanillin, Ferulic acid, Naringenin, Propyl Gallate, Querectin and Cinnamic acid (Fig. 1& Table 2) that might have been responsible for their therapeutic potential. The amounts and structures of polyphenols are shown in Table 2 & Fig. 2.

**Table 2:** Polyphenolic compounds of *D. tortuosa* aerial parts.

Compound	Conc. (µg/g)	Compound	Conc. (µg/g)
*Gallic acid*	92.67	*Coumaric acid*	7.22
*Chlorogenic acid*	41.86	*Vanillin*	13.25
*Catechin*	0.00	*Ferulic acid*	5.43
*Caffeine*	11.79	*Naringenin*	537.70
*Caffeic acid*	9.28	*Propyl Gallate*	1344.46
*Syringic acid*	15.48	4′*.7-Dihydroxy isoFlavone*	0.00
*Rutin*	80.51	*Querectin*	29.37
*Ellagic acid*	11.34	*Cinnamic acid*	8.28

### Characterization of green synthesized ZnO.NPs

During synthesis, the change in color of the solution and formation of a yellowish-white precipitate was an indication that zinc nitrate had been reduced (Fig. S1).

#### U.V. spectrophotometric analysis

The formation of ZnO.NPs was initially confirmed by UV-vis spectroscopy within the range 200–800 nm. The absorption spectrum of green synthesized ZnO.NPs showed a characteristic peak at 374 nm (Fig. S2). The direct band gap (E_g_) calculated was 3.32 eV.

#### FTIR spectroscopic analysis

FTIR is used as a confirmatory technique to the nanoparticle formation and offers an impression to the vibrational and rotational modes of the existing molecules, hence it helps to identify the functional and possible phytochemical molecules involved in the reduction and stabilization of ZnO.NPs. Fig. S3 representing the FTIR spectra of ZnO.NPs synthesized by the green approach showed a peak at 442 cm^−1^ which is corresponding to the hexagonal ZnO symmetric bending vibration and a peak at 878 cm^−1^ due to weak vibration of ZnO. The wide peaks present at 3434 cm^−1^ and 1117 cm^−1^ reflect the presence of OH and C—OH stretching vibrations respectively. Other smaller bond vibration peaks including 2925.48 cm^−1^ which denotes the C—H stretching vibration, 2352.73, 1630.52 and 1445.39 cm^−1^ are due to the presence of primary and secondary amines that are characteristics of proteins/enzymes and C–O stretching regions of polysaccharides and phenolic groups.

#### XRD analysis

**Figure 3:**
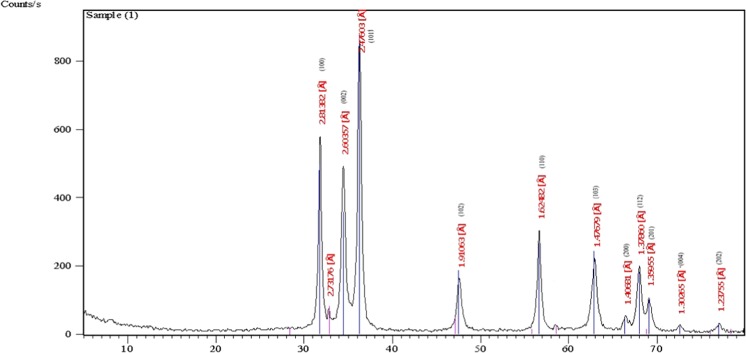
X-ray diffraction pattern of ZnO.NPs.

X-ray diffraction pattern of synthesized ZnO.NPs was obtained as displayed in Fig. 3. The crystalline peaks positioned at (2θ) peaks angles of 31.80°, 34.45°, 36.28°, 47.59°, 56.65°, 62.94°, 66.46, 68.00°, 69.09, 72.57 and 77.0648° correspond to the reflection from (100), (002), (101), (102), (110), (103), (200), (112), (201), (004) to (202) crystal planes, respectively. By using Scherrer’s formula, the average crystallite size of ZnO which is derived from the FWHM of more intense peak corresponding to 101 plane located at 36.28° was estimated to be 15.41 nm.

#### HR-TEM analysis

**Figure 4:**
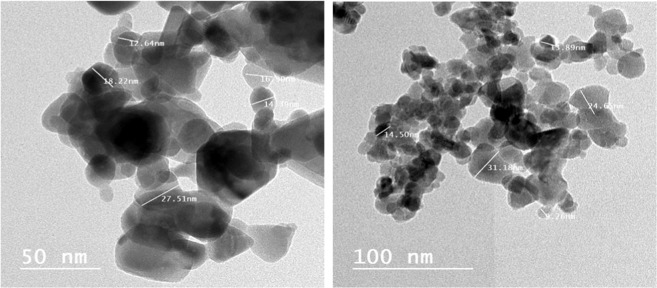
HR-TEM micrograph of ZnO.NPs.

TEM analysis can be used to understand the crystalline characteristics and size of the synthesized NPs. The analysis was carried out using JEOL-2100 and the images at different magnification (50 and 100 nm) are shown in Fig. 4. TEM images displaying the major series of particle size that were between 9.26 to 31.18 nm.

### Cytotoxic assay

**Figure 5:**
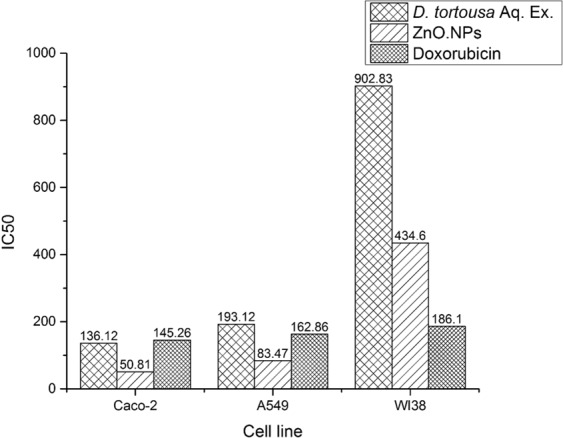
IC_50_ of *D. tortuosa* aqueous extract, ZnO.NPs and Doxorubicin on the Caco-2 and A549 cancer cells compared to WI38 normal cells.

The cytotoxicity of the tested materials (*D. tortuosa* Aq. Ex. and ZnO.NPs) was investigated using Doxorubicin as a positive control, and the untreated cells were the negative control. From the MTT assay results present in Fig. 5, both the plant extract and ZnO.NPs showed a profound selective cytotoxic effect on the Caco-2 and A549 cancer cell lines with appreciable lower cytotoxic activity on the normal WI38 cells. This effect was concentration-dependent, where 1000 μg/ml had a larger effect than 500 μg/mL and so on. Besides, it was obvious that the extent of cytotoxicity influenced by the cell type and the material used. Markedly the ZnO.NPs had the most potent cytotoxic activity and Caco-2 was more sensitive than A549, as shown in Fig. 5. IC_50_ of A549 cells were 193.12 and 83.47 while, IC_50_ values of Caco-2 cells were 136.12 and 50.81 μg/mL by the extract and ZnO.NPs respectively. Significantly higher IC_50_ values 902.83 and 434.60 μg/ml obtained from the treatment of the normal lung epithelial cell (WI38) with the respective materials. While, nearly close IC_50_ values were observed among the different cell lines treated with Doxorubicin 145.26, 162.86, 186.10 μg/ml of Caco-2, A549 and WI38 cell lines, respectively. This is consistent with the microscopic examination data through which the cytotoxic activity could be distinguished by membrane blebbing, cell swelling or shrinkage, nuclear margination or fragmentation, and chromatin condensation (Figs. S4–S6).

## Discussion

Plants considered an inimitable source for the future novel medicine and this value was found to be correlated with their active biocomponents^32^. In this regard, several phytoconstituents were identified in *D. tortuosa* aerial parts. Some important phenolic compounds were quantified with high prevalence of propyl Gallate and Naringenin. Thanks to their functional (hydroxyl) group, phenolics have been informed to be effective hydrogen donors which accounts for various biological activities^33^.

Based on the ability of plants to bioaccumulate metal ions and the involvement of active phytoconstituents as bioreductants and stabilizers, the green synthesis of ZnO.NPs could be illustrated^34^. The reduction of Zn nitrate into ZnO.NPs, which informed through the change in color, may be attributed to excitation of surface plasmon vibrations of nanoparticles which results in Surface Plasmon Resonance^35^.

According to previous studies suggested that ZnO.NPs exhibit a characteristic broad absorption peak between 330–460 nm^36,37^, peaking at 374 nm without any other peaks confirms the synthesis of pure ZnO.NPs with the aid of active biomolecules in the plant extract in reduction and stabilization of synthesized nanoparticles. This absorption peak could be attributed to the intrinsic band-gap absorption of ZnO due to the electron transitions from the valence band to the conduction band (O2p→Zn3d) as explained by Zak *et al*.^38^. Absorption in this wavelength of 374 nm further confirms that the absorption spectrum is slightly blue-shifted with respect to the bulk value (377 nm) of the ZnO NPs. This blue shift in the absorption edge is due to the quantum confinement effect among the individual nanoparticles. The good absorption of the ZnO-NPs in the UV region implies its applicability in the medical application such as sunscreen protectors or in antiseptic ointments^39^. The band gap and catalytic activity of metal oxide nanoparticles play a key role in their cytotoxic response to biological systems^40,41^.

From the FTIR results, appearance of other peaks may indicate the existence of enzymes, proteins and metabolites such as alkaloids, flavonoids, polyphenols and carboxylic acid “which remained bound to ZnO.NPs despite repeated washing”. These compounds, particularly flavonoids and other phenolics, assisted in the reduction of zinc ions to ZnO.NPs. The stability of the synthesized ZnO.NPs could presumably be accounted for the presence of free amino and carboxylic groups that have interacted with the zinc surface. Furthermore, the proteins present in the medium help in the stabilization of ZnO.NPs by forming a coat, covering the metal nanoparticles and preventing the nanoparticles agglomeration^35,42^ (Fig. S3).

All diffraction peaks of the sample obtained from XRD indicated the purity of ZnO nanocrystalline formation. In accordance with the JCPDS 36–1451card^43^, identical to the hexagonal phase with Wurtzite structures. The calculated particle size was found to lie within the size range of 9.26 to 31.18 nm obtained by TEM.

TEM images confirmed the hexagonal structure of the synthesized ZnO.NPs. This structure implies more ionicity and subsequent enhanced catalytic activity of the NP among the three 2D ZnO.NPs structures^44^. It is well documented that decreasing of particle size increases their functionality as antimicrobial and anticancer agent due to the larger surface-to-volume ratio^45^.

Cancer, the malignant case of uncontrolled cell proliferation, considered the second leading cause of death worldwide. Among the different cancer types; colorectal and lung cancers are alarming and being associated with the greatest mortality^46^. One of the most challenging fields in modern scientific research is developing new anticancer drugs with minimal side effects and enhanced selectivity and efficacy^47^. Ethno-medicine is as ancient as human civilization and many plant extracts and phytoconstituents have been identified to have antioxidant and anticancer properties. Earlier to the current results, Abdallah and Ezzat (2011), notified a similar cytotoxic activity of *D. tortuosa* essential oil against liver (HEPG2), colon HCT116), and breast cancer (MCF7). They related this activity to the high contents of terpinen-4-ol, γ-terpinene, sabinene, and β-myrcene^13^. Furthermore, phenols and polyphenolic compounds were found to be associated with the inhibition of cancer and atherosclerosis^33,48^. The phytoconstituents could exert their activity against carcinogens through the production of the Reactive Oxygen Species (ROS) which are involved in phagocytosis, regulation of cell proliferation and intracellular signaling^49^. On the other hand, many nano-medicine researches stated that ZnO nanostructures can be utilized to fight cancer cells, providing a possible target for the development of anti-tumor agents^50,51^ which support our observation that ZnO.NPs are dramatically less toxic to normal cells. Our finding that cytotoxicity depended on the cell type and the material used comes in full agreement with the previous studies^13,52^. Moreover, the present results confirmed the previously reported strong preferential cytotoxicity of ZnO.NPs to cancerous cells. A similar trend of selective cytotoxicity against three types of cancer cells, human hepatocellular carcinoma HepG2, human bronchial epithelial BEAS-2B and human lung adenocarcinoma A549 was reviewed by Akhtar *et al*.^53^. Likewise, ZnO.NPs exhibited a significant cytotoxic effect on HEp-2 cells as proclaimed by Jacob *et al*.^54^. Even though, the precise mechanism of cytotoxic activity of ZnO.NPs is yet under debate, several proposed ones are suggested and adopted. The basic mechanism behind the cytotoxicity of ZnO.NPs is the intracellular release of dissolved zinc ions, accompanied by ROS induction. This action is induced through a binary response: comprising the pro-inflammatory reaction of the cell against ZnO.NPs in addition to the characteristic surface property of the nanoparticle that makes ZnO.NPs act as a redox system^24,55^.

## Conclusion

In this work, the aqueous extract of *Deverra tortuosa* was used for the green synthesis of ZnO nanoparticles. The phytochemical screening of *D. tortuosa* extract revealed copious kinds of active constituents with a high polyphenolic content that have the ability to chelate metal ions and aid in the bioreduction of ZnO.NPs. This promotes its utilization for the green synthesis of other metal NPs (e.g. Ag, Au). Moreover and to the best of our knowledge, still no published reports have investigated using *D. tortuosa* aqueous extract against the cancer cell lines (Caco-2 and A549). Different techniques were used for authenticating ZnO nanoparticles “UV–visible, FTIR, XRD and TEM” that confirmed the presence of nanoparticles with an average size of 15.22 nm. Both the aqueous extract of *D. tortuosa* and the synthesized ZnO nanoparticles showed an attractive selective cytotoxic activity against two tested cancer lines, offering satisfying ‘safer and cheaper’ alternatives to conventional therapy protocols. With all their promising characteristics of green synthesized ZnO.NPs, other biological activities should be evaluated. Moreover, the results of the current study need to be corroborated by testing these materials for their *in vivo* application.

## Supplementary information

Supplementary information.
