# Supplementary material for: Green Synthesis of Zinc Oxide Nanoparticles Using Aqueous Extract of Deverra tortuosa and their Cytotoxic Activities
Source: Sci Rep. 2020 Feb 26;10:3445. doi: 10.1038/s41598-020-60541-1 (PMC7044426; doi:10.1038/s41598-020-60541-1)
Supplement: Supplementary file 1 — Supplementary information. [file 41598_2020_60541_MOESM1_ESM.docx]

**Supplementary information:**

**Green Synthesis of Zinc Oxide Nanoparticles Using Aqueous Extract of *Deverra tortuosa* and their Cytotoxic Activities**

**Yasser A. Selim^a,^** *****, Maha A. Azb^b^ , Islam Ragab^c^ and Mohamed H. M. Abd El-Azim^d^**

*^a^Faculty of Specific Education, Zagazig University, Zagazig 44519, Egypt,* [*y2selem@yahoo.com*](mailto:y2selem@yahoo.com)

*^b^ Botany Department, Faculty of Science, Zagazig University, Zagazig 44519, Egypt,* [*res.mahaadel@yahoo.com*](mailto:res.mahaadel@yahoo.com)

*^c^Faculty of Science and Arts, Riyad Alkhabra, Qassim University, Qassim, Saudi Arabia,* [*chem_islam82@yahoo.com*](mailto:chem_islam82@yahoo.com)

*^d^Chemistry Department, Faculty of Science, Zagazig University, Zagazig 44519, Egypt,* [*drmhm1982@yahoo.com*](mailto:drmhm1982@yahoo.com)

*Corresponding author: E-mail: [y2selem@yahoo.com](mailto:y2selem@yahoo.com)

**Abstract:**

In recent years, there is a growing interest towards the green synthesis of metal nanoparticles, particularly from plants; however, still no published data on the synthesis of ZnO.NPs using the *Deverra tortuosa* extract. Through this study, zinc oxide nanoparticles (ZnO.NPs) have been synthesized based on using the environmentally benign extract of the aerial parts of *D. tortuosa* as a reducing and capping agent. ZnO.NPs synthesis was confirmed using UV-Visible (UV-Vis) spectroscopy, Fourier Transform Infrared Spectroscopy (FTIR), X-ray Diffraction (XRD) and High Resolution-Transmission Electron Microscope (HR-TEM). The qualitative and quantitative analyses of plant extract was done. The potential anticancer activity was *in vitro* investigated against two cancer cell lines (human colon adenocarcinoma “Caco-2” and human lung adenocarcinoma “A549”) compared to their activities on the human lung fibroblast cell line (WI38) using the MTT assay. Both the aqueous extract and ZnO.NPs showed a remarkable selective cytotoxicity against the two examined cancer cell lines.

**Keywords**: *Deverra tortuosa*; ZnO nanoparticles; antioxidant activity; anticancer activity

**Table S1:** d-spacing calculations for ZnO.NPs and reflection planes

| No. | h | k | l | Pos. [°2Th.] | θ | Sin θ | d-_XRD_ spacing [Å] | d-_JCPDS_  spacing [Å] | Intensity (%) |
| --- | --- | --- | --- | --- | --- | --- | --- | --- | --- |
| 1 | 1 | 0 | 0 | 31.8028 | 15.9014 | 0.2739 | 2.81382 | 2.184 | 65.18 |
| 2 | 0 | 0 | 2 | 34.448 | 17.224 | 0.2961 | 2.60357 | 2.6034 | 54.94 |
| 3 | 1 | 0 | 1 | 36.2824 | 18.1412 | 0.3114 | 2.47603 | 2.4757 | 100 |
| 4 | 1 | 0 | 2 | 47.5941 | 23.79705 | 0.4035 | 1.91063 | 1.9111 | 17.32 |
| 5 | 1 | 1 | 0 | 56.6503 | 28.32515 | 0.4745 | 1.62482 | 1.6250 | 33.94 |
| 6 | 1 | 0 | 3 | 62.9379 | 31.46895 | 0.522 | 1.47679 | 1.4773 | 23.76 |
| 7 | 2 | 0 | 0 | 66.4603 | 33.23015 | 0.548 | 1.40681 | 1.4071 | 4.67 |
| 8 | 1 | 1 | 2 | 68.0032 | 34.0016 | 0.5592 | 1.3786 | 1.3784 | 21.92 |
| 9 | 2 | 0 | 1 | 69.09 | 34.545 | 0.567 | 1.35955 | 1.3584 | 11.18 |
| 10 | 0 | 0 | 4 | 72.573 | 36.2865 | 0.5918 | 1.30265 | 1.3017 | 2.11 |
| 11 | 2 | 0 | 2 | 77.0648 | 38.5324 | 0.623 | 1.23755 | 1.2378 | 3.05 |


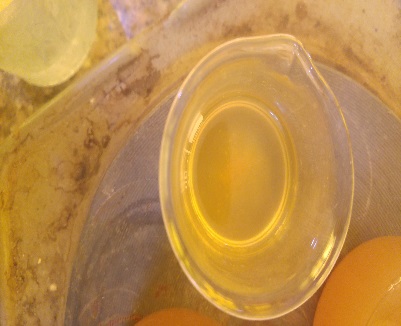

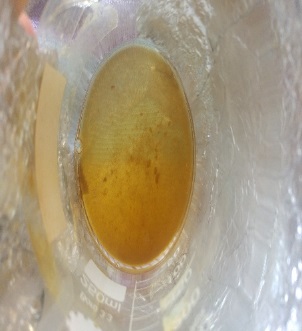


**Figure S1:** The initial change in color during green synthesis of ZnO.NPs


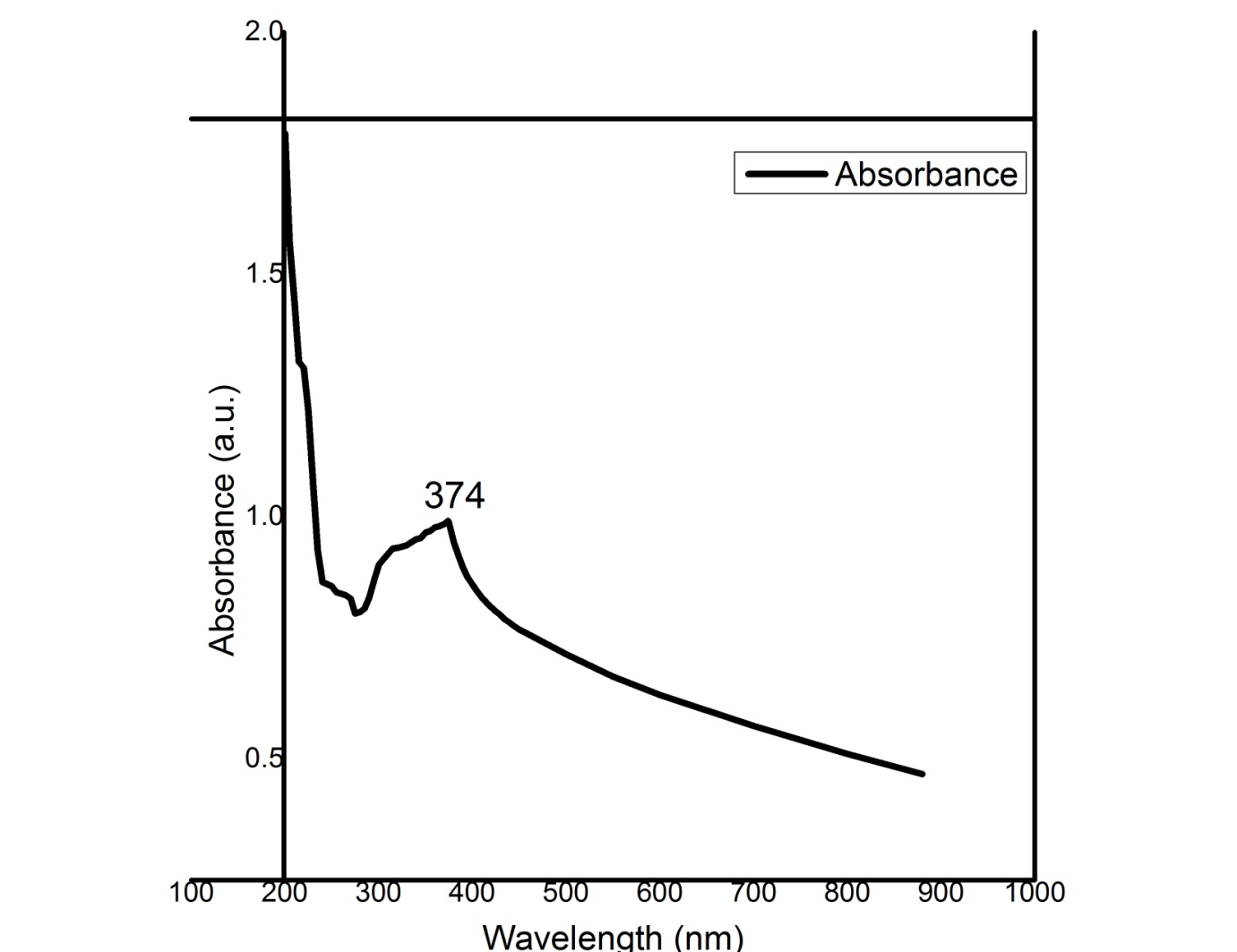


**Figure S2:** The UV-vis spectroscopy spectra of ZnO.NPs


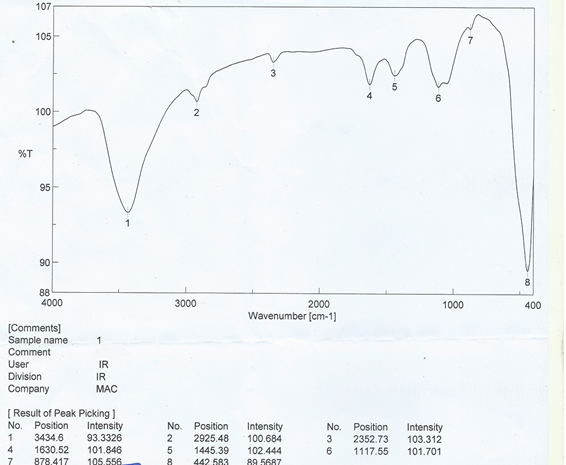


**Figure S3:** The Fourier transform infrared spectroscopy pattern of ZnO.NPs


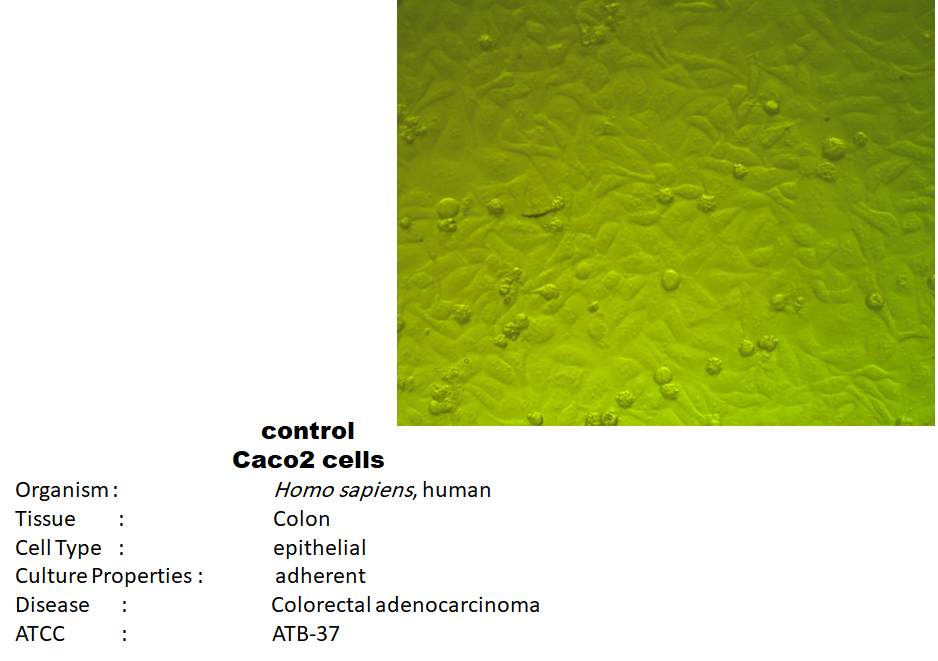


**A**


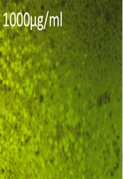

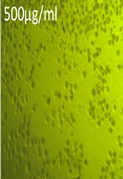

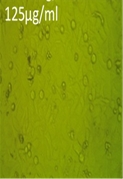

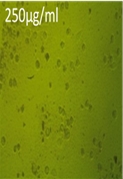

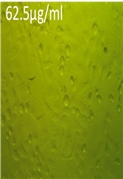

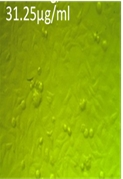


**1**


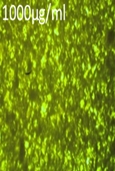

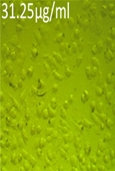

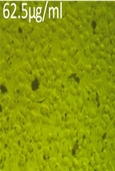

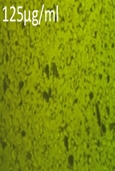

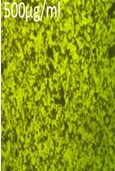

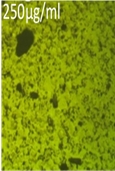


**2**


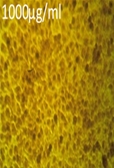

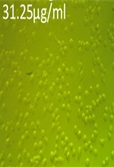

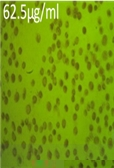

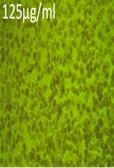

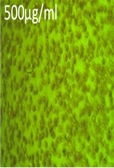

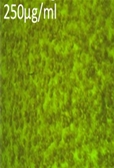


**3**

**Figure S4:** Morphological Examination of A) Caco-2 adenocarcinoma cell line after thetreatment with**: 1)** *D. tortuosa* Aqueous Extract; **2)** ZnO.NPs; **3)** Doxorubicin


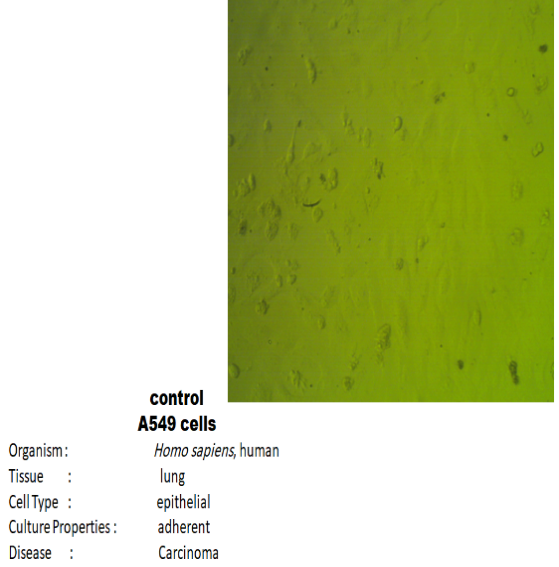


**B**


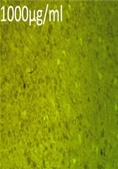

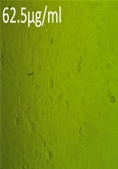

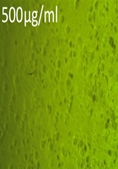

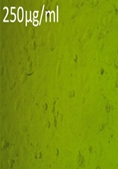

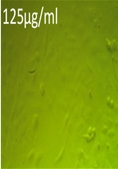

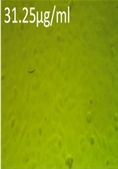


**1**

**3**


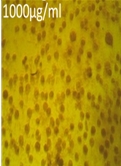

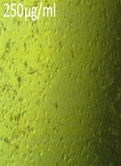

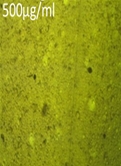

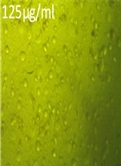

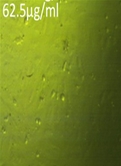

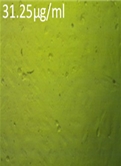


**2**


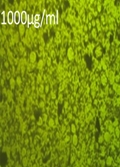

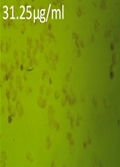

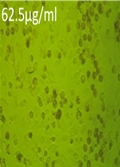

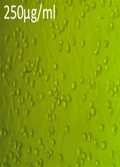

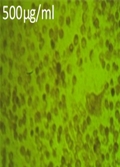

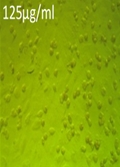


**Figure S5:** Morphological Examination of B) A549 adenocarcinoma cell line after thetreatment with**: 1)** *D. tortuosa* Aqueous Extract; **2)** ZnO.NPs; **3)** Doxorubicin


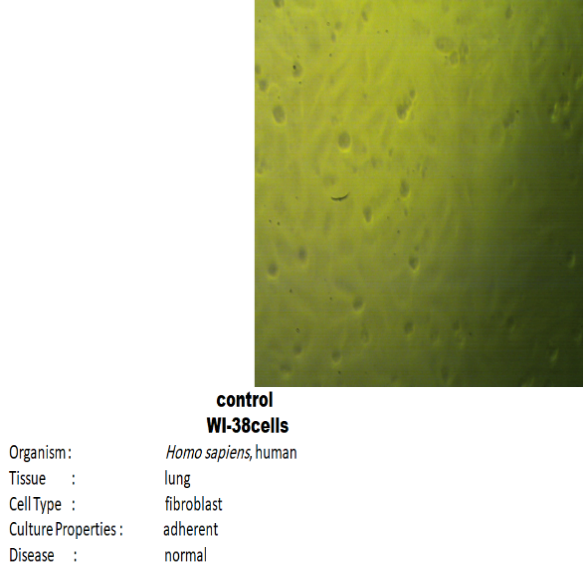


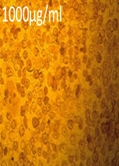

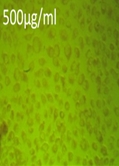

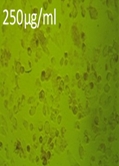

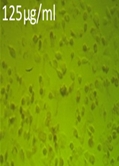

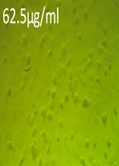

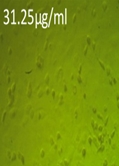


**3**

**Figure S6:** Morphological Examination of C) WI38normal cell line after thetreatment with**: 1)** *D. tortuosa* Aqueous Extract; **2)** ZnO.NPs; **3)** Doxorubicin


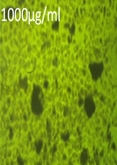

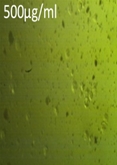

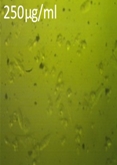

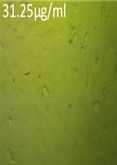

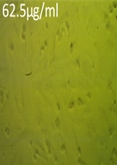

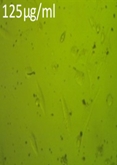


**2**


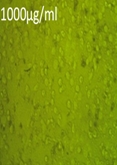

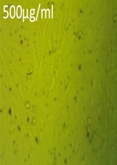

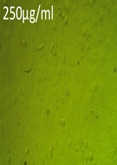

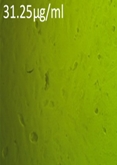

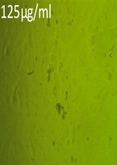

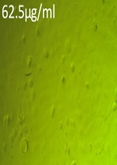


**1**

**C**
